# Patellar Shape Variation in Cats and Dogs: Implications for Orthopedic Surgical Planning

**DOI:** 10.3390/ani15111608

**Published:** 2025-05-30

**Authors:** Ebru Eravci Yalin, Yusuf Altundağ, Kemal Altunatmaz, Buket Çakar, Esra Acar, Edyta Pasicka, Ermiş Özkan, Ozan Gündemir, Mihaela-Claudia Spataru

**Affiliations:** 1 Department of Surgery, Faculty of Veterinary Medicine, Istanbul University-Cerrahpasa, Istanbul 34320, Türkiye; ebrueravci@gmail.com; 2 Department of Surgery, Faculty of Veterinary Medicine, Namik Kemal University, Tekirdag 59030, Türkiye; yaltundag@nku.edu.tr; 3 VetAmerican Animal Hospital, Istanbul 34406, Türkiye; kemal.altunatmaz@iuc.edu.tr; 4 Institute of Graduate Studies, Istanbul University-Cerrahpasa, Istanbul 34320, Türkiye; buket.cakar@ogr.iuc.edu.tr (B.Ç.); esra.acar.vet@gmail.com (E.A.); 5 Department of Biostructure and Animal Physiology, Faculty of Veterinary Medicine, Wrocław University of Environmental and Life Sciences, 50-375 Wroclaw, Poland; edyta.pasicka@upwr.edu.pl; 6 Department of Anatomy, Faculty of Veterinary Medicine, Istanbul University-Cerrahpasa, Istanbul 34320, Türkiye; ermisozkan@iuc.edu.tr; 7 Department of Public Health, Faculty of Veterinary Medicine, “Ion Ionescu de la Brad” Iasi University of Life Sciences, 700489 Iasi, Romania; mspatarufmv@yahoo.com

**Keywords:** 3D modeling, allometry, geometric morphometrics, shape variation, orthopedic surgery

## Abstract

The patella plays a vital role in stifle joint function by contributing to limb extension and joint stabilization. While its clinical significance in small animal orthopedic surgery is well established, detailed comparative data on patellar morphology in domestic cats and dogs remain limited. This study aimed to investigate interspecific and intraspecific variation in patellar shape and size using three-dimensional geometric morphometric techniques. Computed tomography images of 18 cats and 55 dogs were used to construct 3D models, and a total of 14 anatomical landmarks were manually placed on each patella. Generalized Procrustes Analysis was applied, followed by principal component analysis to explore patterns of shape variation. The results revealed a significant difference in shape between cats and dogs, with dogs exhibiting broader variation and larger centroid sizes. Regression analysis indicated that 12.2% of the observed shape variation could be attributed to centroid size, reflecting the influence of patellar size. This strong link suggests that size has a significant influence on how the patella’s shape varies, especially in dogs. These findings highlight the importance of individual variation in patellar morphology, particularly in dogs. A single standardized implant or surgical technique may not be appropriate for all cases. Integrating shape and size evaluations into preoperative imaging can help improve implant fit, joint stability, and overall surgical success. In the absence of breed-specific data, individualized planning remains the most reliable approach.

## Simple Summary

In veterinary surgery, especially when treating injuries such as patellar fractures or deformities like medial patellar luxation, understanding the shape and size of the patella (kneecap) is essential. However, detailed comparisons of patellar shape and size in common pet animals such as cats and dogs remain limited. The results showed that cats tend to have more uniform patellar shapes, while dogs exhibit a wider range of variation in both shape and size. This diversity may be related to the large number of dog breeds and their differing body structures. The findings indicate that a single surgical approach—such as patellar fracture fixation or trochlear groove reconstruction—or a standardized implant design like fixation plates may not be suitable for all individuals. Instead, preoperative planning, including the selection of surgical technique and implant type, based on the specific shape of each animal’s patella may improve surgical success. These results could support veterinarians in making more informed decisions—such as selecting appropriate surgical techniques or implant designs tailored to the patella’s morphology—and may contribute to the development of improved orthopedic tools (e.g., size-adaptable implants) and more effective treatments for joint-related conditions like patellar luxation or fracture repair in pets.

## 1. Introduction

The patella, commonly known as the kneecap, is a sesamoid bone that articulates with the femur and plays a critical role in protecting the anterior articular surface of the knee joint [1]. It serves as a key component of the extensor mechanism, which consists of the quadriceps muscles, the patella itself, and the patellar tendon connecting to the tibial crest [2,3]. This structure allows for smooth flexion and extension of the stifle joint as the patella glides within the femoral trochlear groove [4,5]. The bone is present in many tetrapods, including mammals and birds, and exhibits species-specific morphological differences [6,7]. In domestic dogs, the patella is typically almond-shaped and relatively symmetrical in terms of width and depth [8]. It is biomechanically significant in various orthopedic conditions, and recent studies have demonstrated the effectiveness of advanced fixation techniques, such as locking plates and screws, for treating patellar fractures [9,10,11]. In contrast, the feline patella is smaller and more delicate, rendering certain surgical interventions—such as screw placement or drilling—risky and potentially harmful [12,13]. Despite the clinical relevance of these species-specific differences, detailed morphometric comparisons of the patella in cats and dogs remain limited in the literature. A clearer understanding of patellar shape variation—both between and within species—could enhance surgical planning, implant design, and clinical decision-making in small animal orthopedics.

Geometric morphometrics is a powerful analytical method that enables the quantitative investigation of shape differences among biological structures [14,15,16]. This technique goes beyond traditional morphometric measurements by allowing for detailed comparisons of shape variation through anatomically defined landmark points within a three-dimensional coordinate system [17,18,19]. It is widely used in the evaluation of concepts such as shape asymmetry, allometry, and evolutionary adaptation, particularly in surgical anatomy, functional morphology, and interspecies comparative studies [20,21,22]. Today, geometric morphometrics has become a crucial tool not only in fundamental research within veterinary morphology but also in shaping clinical applications.

In recent years, the integration of geometric morphometric methods with advanced imaging systems has contributed significantly to shape-based approaches in veterinary surgical science [23]. For instance, a study analyzing turtle shell morphology using geometric morphometrics reported that shape variations observed among individuals revealed important differences that could be considered in treatment planning [24]. Similarly, in another study, preoperative and postoperative imaging data were compared to evaluate shape changes in bone structure throughout the healing process [25].

The patella is a sesamoid bone that plays a crucial role in stabilizing the stifle joint and transmitting the force of the extensor mechanism [2,5]. In small animal orthopedic surgery, the morphology of the patella is of significant clinical relevance, particularly in the diagnosis and treatment of conditions such as patellar luxation, patellofemoral pain syndrome, and traumatic fractures [9,13]. Recent advancements in surgical techniques have introduced sophisticated interventions including trochlear groove reconstruction, osteotomies, and even custom-made 3D-printed implants [26,27,28]. However, the success of these procedures relies heavily on preoperative planning that accounts for individual and species-specific anatomical variations. While several studies have emphasized species-related morphological differences in the femoral trochlea, quantitative three-dimensional analyses of the patella itself remain scarce. In particular, evaluating the patellar shape through interspecies comparisons and assessing its morphological variation in a way that can inform surgical planning are still underexplored areas in the veterinary literature.

The aim of this study is to conduct a comparative assessment of patellar morphology in domestic cats and dogs using three-dimensional geometric morphometric techniques. The central research question addresses whether patellar shape significantly differs between these two species, and whether the observed variation—both between and within species—has potential implications for procedures such as patellar luxation repair, trochleoplasty, or fracture fixation, where congruency between the patella and the femoral trochlea is essential for surgical success. In addition, this study examines how shape variation is influenced by allometric relationships—specifically, the effect of patellar size (centroid size) on shape variation—as well as by morphometric parameters such as centroid size and body weight. Through this approach, we aim to provide a scientific foundation that supports individualized surgical planning and highlights the need to consider morphological variation when approaching patellar interventions in clinical practice.

## 2. Materials and Methods

### 2.1. Animals

A total of 73 animals, including 18 cats and 55 dogs, were used in this study. Only the right patella of each animal was included in the analysis. All specimens were confirmed to be free of any pathological conditions affecting the patella. For each animal, sex, age, and body weight were recorded. Written informed consent was obtained from the owners of the animals. Ethical approval for this study was granted by the Local Ethics Committee for Animal Experiments of Istanbul University-Cerrahpaşa (IUC-HADYEK).

The computer tomography images used in this study were obtained retrospectively from the imaging archive of the Animal Hospital at the Faculty of Veterinary Medicine, Istanbul University-Cerrahpaşa. Scans were performed using Siemens Somatom Scope vc30b (Siemens, Munich, Germany) and Siemens Somatom Sensation 16 systems (Siemens, Munich, Germany). The scanning protocol was standardized across all samples with the following parameters: 0.6 mm slice thickness, 110 kV voltage, 28 mA current, and a total scan time of approximately 14 s per sample.

### 2.2. Three-Dimensional Modeling and Landmarking

**Figure 1:**
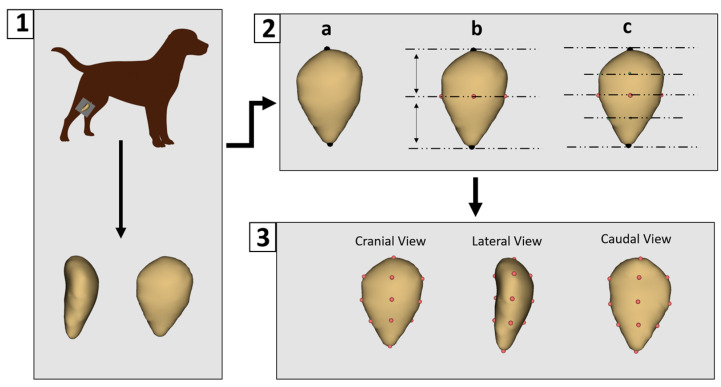
Three-Dimensional modeling and landmark placement process. (**1**) Patellar images were obtained using computed tomography and three-dimensional models were generated. (**2**) Manual digitization began with the placement of two primary landmarks: one at the most superior point of the patella and the other at its central point (**a**). Intermediate landmarks were then placed at the anterior, posterior, medial, and lateral midpoints between these primary landmarks (**b**). Additional landmarks were placed halfway between each primary and intermediate point (**c**). (**3**) This resulted in a total of 14 consistently applied landmarks for each patella model.

All CT data were processed using 3D Slicer software (version 5.8.1) for segmentation [29]. Non-osseous (soft) tissues were removed to isolate the patella bone. Each sample was then saved as a three-dimensional surface model in “.ply” format. Landmarking was performed using 3D Slicer (version 5.8.1). A preliminary landmarking scheme was developed and subsequently applied consistently across all specimens by a single researcher to ensure reproducibility and standardization. A total of 14 landmarks were placed on each patella model (Figure 1).

The patella lacks distinct anatomical features that can be reliably used for automatic landmark placement. Although automated landmarking was initially tested, inconsistencies were observed between corresponding points, leading to the decision to perform manual landmarking instead. Manual digitization began with the placement of two primary landmarks: one at the most superior point of the patella and another at its central point. Subsequently, intermediate landmarks were placed at the anterior, posterior, medial, and lateral midpoints between these primary landmarks. Additional landmarks were then added midway between the primary and intermediate points, resulting in a total of 14 landmarks being consistently applied to each patella model.

### 2.3. Geometric Morphometrics and Statistical Analysis

**Figure 2:**
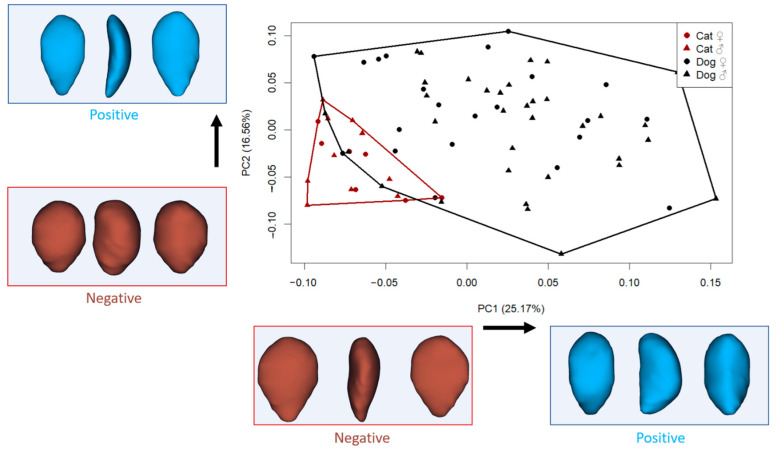
Principal component analysis (PC1–PC2) and visualization of shape variation (cranial, lateral, and caudal view). (Each dot represents one specimen plotted according to its shape coordinates along the first two principal components (PC1 and PC2). The axes represent directions of major shape variation: PC1 explains the greatest amount of variation, while PC2 explains the second most. Shape deformation diagrams at the ends of each axis help interpret what kind of morphological changes are associated with positive or negative values. For example, moving from left to right along PC1 corresponds to a transition from a triangular to a more rectangular patellar shape).

All analyses were conducted using the Geomorph package (v.4.0.9) in R (RStudio 2024.09.1) [30]. Generalized Procrustes Analysis (GPA) aligns the 3D coordinates of anatomical landmarks by removing differences in position, scale, and orientation so that only shape differences remain. Centroid size (CS) is a measure of the overall size of the object based on the dispersion of landmarks around the centroid. Principal component analysis (PCA) is then used to reduce the high-dimensional shape data into a smaller number of principal components (PCs) that capture the most important patterns of variation. The Geomorph package in R performs these steps and generates scatterplots (such as Figure 2) that visually summarize shape variation across specimens.

To evaluate the effect of size on shape, a multivariate regression of shape coordinates on log-transformed centroid size was conducted, serving as a test for allometry. In order to assess whether patellar shape significantly differed between cats and dogs, statistical comparisons were made using the first principal component (PC1), which accounted for the largest portion of shape variation. A one-way analysis of variance (ANOVA) was used to test for significant differences in PC1 scores between the two species.

## 3. Results

### 3.1. Size

To assess differences in overall patellar size between cats and dogs, centroid size was calculated for each specimen. Cats exhibited a relatively consistent patella size, with a mean CS of 18.28 ± 1.61. In contrast, dogs showed a markedly greater size variation, with a higher mean CS of 22.56 ± 7.01. A one-way ANOVA revealed that the difference in mean centroid size between species was statistically significant (F = 6.20, *p* = 0.015). The broader range of patellar sizes in dogs likely reflects greater intraspecific diversity, potentially linked to breed differences, or body size variability. This contrasts with the relatively constrained size profile observed in cats.

### 3.2. Shape Variation

The principal component analysis revealed distinct patterns of shape variation in the patellae of cats and dogs (Figure 2). The first principal component, which accounted for 25.17% of the total shape variation, primarily captured differences in patellar outline, ranging from slender, triangular shapes to broader, more rectangular and thickened forms. Specimens with negative PC1 scores exhibited a more triangular patella shape, where the upper half was broader than the lower portion, resulting in a more elongated and mediolaterally narrower distal region. In contrast, positive PC1 scores were associated with a more rectangular shape, with increased thickness and a more uniform vertical profile.

The second principal component (PC2), explaining 16.56% of the variation, described shape changes associated with the roundness and tapering of the patella. Negative PC2 values were linked to a thick and oval patella structure, while positive PC2 scores indicated a thinner, more triangular shape, particularly with a broader upper region.

In the morphospace defined by PC1 and PC2, some small-sized dogs clustered near the cat group, suggesting partial shape overlap among similarly sized individuals despite species differences.

We aimed to evaluate whether the primary axis of shape variation (PC1) significantly distinguished between cats and dogs. The results revealed a highly significant difference in PC1 scores between the two species (F: 38.21, *p* < 0.001). This indicates that PC1 captures a major shape distinction related to species identity. As described earlier, specimens with negative PC1 values displayed a more triangular and slenderer patella, with a broader upper half relative to the lower, while specimens with positive PC1 values exhibited a more rectangular and thicker patellar shape.

### 3.3. Allometry

**Figure 3:**
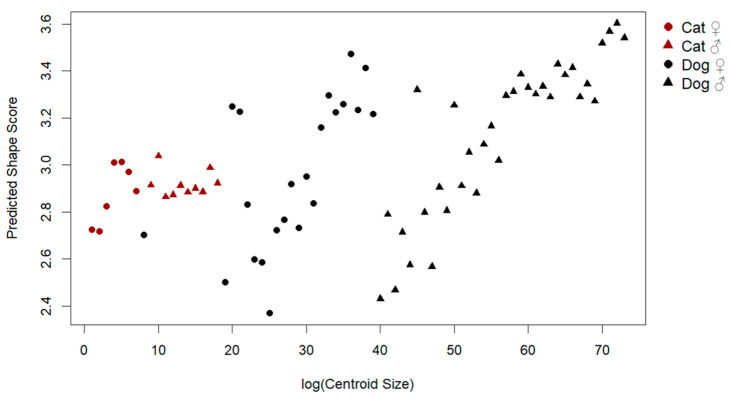
Allometric relationship between log centroid size and predicted shape scores.

To examine the influence of size on patellar shape, a multivariate regression of shape on log-transformed centroid size was conducted (Figure 3). The analysis revealed a significant allometric effect, with size explaining 12.2% of the total shape variation (Rsq: 0.122, F: 9.87, *p*: 0.001). Visual inspection of the allometric regression demonstrated consistent shape changes with increasing size, including shifts from a more triangular and slender morphology to a broader and thicker patellar outline.

**Figure 4:**
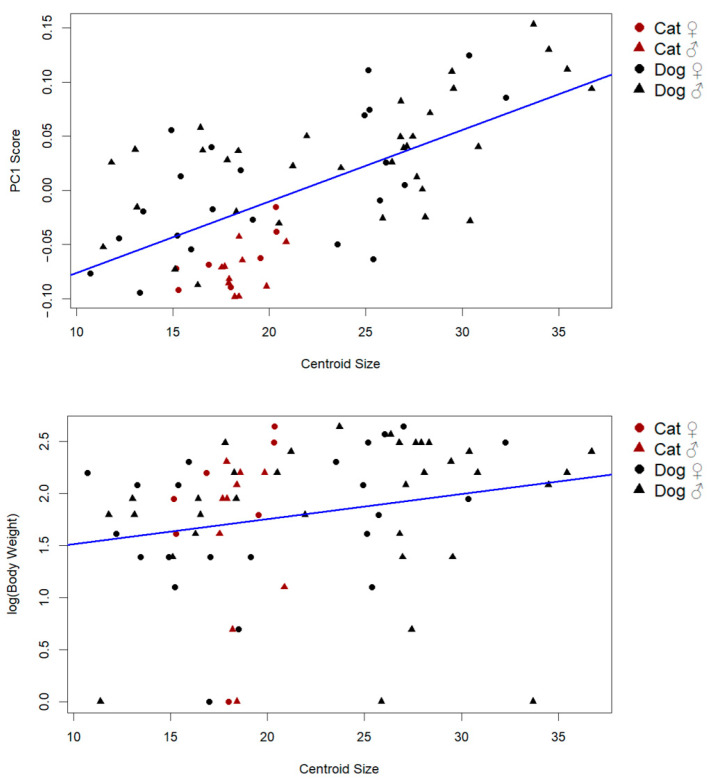
Influence of PC1 scores and log body weight on centroid size (the predicted shape refers to the estimated Procrustes coordinates resulting from the regression model, representing the expected shape corresponding to a given centroid size. These predicted shapes were used for visualization of allometric trajectories).

Supporting this result, a strong positive correlation was found between centroid size and PC1 scores (r = 0.65, *p* < 0.001), indicating that the primary axis of shape variation includes a substantial allometric component (Figure 4). This suggests that larger individuals tend to exhibit predictable and directional shape changes, consistent with allometric scaling.

By contrast, the relationship between body weight and centroid size was weak and only marginally significant (r: 0.21, *p*: 0.069), implying that while weight may contribute slightly to size variation, it is not the primary driver. These findings collectively demonstrate that size-related shape variation in the patella is largely governed by allometric effects rather than mass alone (Figure 4).

## 4. Discussion

This study provides the first detailed three-dimensional geometric morphometric comparison of patellar morphology between domestic cats and dogs. The findings clearly demonstrate that patellar shape and size differ significantly between the two species, with dogs exhibiting both larger average centroid sizes and a markedly broader range of shape variation. These results are clinically relevant, particularly in the context of orthopedic surgeries such as patellar luxation repair, trochleoplasty, and fracture fixation, where patellar shape can influence implant positioning and joint congruency. The findings may also inform the design and selection of size-specific implants like fixation plates and trochlear wedges. A significant difference was observed in PC1 scores (F = 38.21, *p* < 0.001), which effectively separated cat and dog specimens based on patellar outline geometry. This supports the idea that patellar morphology is not only species-dependent but also provides distinctive shape features that allow for reliable differentiation between cats and dogs.

Furthermore, the observed allometric relationship—where size explained over 12% of the shape variation and correlated strongly with PC1—emphasizes that shape differences are partially driven by body size scaling. This relationship was evident in the transition from slender and triangular patellae to broader and more rectangular forms with increasing size. Although body weight showed only a marginal association with size (r = 0.21), this suggests that shape divergence is influenced not only by body mass but also by functional adaptation, where changes in patellar morphology may reflect biomechanical demands associated with size, such as joint loading and locomotor efficiency. The greater shape diversity observed in dogs, indicated by larger convex hull areas and higher variability along principal components, likely reflects the influence of breed-specific selection pressures, musculoskeletal loading, and a broader range of locomotor adaptations.

The observed similarity in patellar shape between small dogs and cats may have practical implications. Despite differences in centroid size, the overlapping shape characteristics suggest that similar surgical techniques or implant designs could potentially be used in small-sized dogs and cats. This convergence might support a unified approach in certain clinical cases, especially when breed-specific implants are unavailable.

These results may support preoperative planning in procedures such as trochlear reconstruction or patellar luxation repair, where the size and shape of the patella should be taken into account to ensure proper joint congruency. A broader, more rectangular patella may require a deeper and wider trochlear groove, while a narrower or triangular patella may be sufficiently stabilized within a shallower groove. Failing to match groove geometry with patellar morphology can lead to joint incongruity, improper tracking, or increased risk of reluxation. In this context, species- or size-specific anatomical data may inform the selection and design of surgical implants such as trochlear wedges, pre-contoured groove prostheses, or 3D-printed cutting guides tailored to individual joint morphology. As highlighted in previous clinical reports, canine patellae may accommodate more robust fixation methods, while feline patellae require more delicate, conservative approaches due to their smaller size and more constrained shape [12,31,32].

The pronounced morphological variation observed in the knee of dogs—both in terms of shape and size—suggests that adopting a uniform approach in orthopedic surgical interventions and implant design may present challenges [26,33]. Compared to cats, the wider range of variation in dogs is likely influenced by the broad diversity of breeds and the resulting anatomical heterogeneity within the species. Commercially produced orthopedic implants, such as fixation plates, synthetic trochlear grooves, or 3D-printed patellar components, are often based on standard morphological templates [34,35]. However, our findings indicate that such standardized designs may not be optimal for all canine patients, particularly those that fall at the extremes of the morphological spectrum. For this reason, implant development for dogs may benefit from incorporating more flexible, morphometric data-informed systems. Options such as breed-specific sizing, modular components, or preoperatively customized implants based on 3D imaging could help improve surgical fit and potentially reduce the risk of complications. Furthermore, integrating geometric morphometric analysis into routine preoperative planning may support more effective and individualized treatment strategies.

Some case-based reports suggest that the medial–lateral width of the feline patella may not always align well with the femoral trochlear groove, potentially affecting surgical outcomes in select individuals [36]. One of the key anatomical factors contributing to such unfavorable results is the high degree of morphological variation among individuals. This issue becomes even more relevant in dogs, which exhibit considerable diversity in both size and biomechanical structure. The present study also revealed substantial interindividual variation in patellar shape. Although PC1 effectively distinguished between species, PC2—accounting for 16.56% of the total shape variation—appeared to reflect intraspecific variation in both cats and dogs. The shape changes associated with PC2 described a transition between flatter and thicker patellar morphologies. These variations directly influence how well the patella conforms to the femoral groove, which is crucial for joint stability. Therefore, the presence of both inter- and intraspecific shape diversity highlights the importance of incorporating individual anatomical assessments into surgical planning involving the patella.

One of the limitations of this study lies in the high morphological variation observed among the canine specimens, which may reflect significant shape differences between dog breeds. However, breed-specific identification was not available for most of the dogs included in this study, and the sample consisted of various unidentified or mixed-breed individuals. As a result, it was not possible to assess breed-related morphological patterns within the canine group. Previous research has demonstrated that skeletal elements such as the calcaneus may show breed-specific morphological characteristics in both cats and dogs. It is therefore plausible that the patella, too, may exhibit shape features that are unique to certain breeds. Future studies that include well-defined breed samples could provide valuable insight into breed-specific patellar morphology and may further enhance the clinical relevance of morphometric findings in orthopedic planning.

Although the role of patellar position in stifle joint stability and pathology is well established in the veterinary literature, the importance of patellar size and shape has received less attention. However, patellar morphology may indirectly influence joint function by affecting trochlear congruency, contact surface area, and mechanical tracking of the extensor mechanism. A mismatched patellar shape may lead to altered load distribution, increased joint stress, or suboptimal articulation, particularly in cases of trochlear dysplasia or patellar luxation. Moreover, in dogs, wide morphological variation—such as that observed in chondrodystrophic breeds or breeds with shallow trochlear grooves—suggests that morphology may play a more relevant role than previously assumed. While direct experimental studies are limited, our morphometric findings provide foundational data to support future biomechanical and clinical investigations addressing how patellar shape may influence joint function.

## 5. Conclusions

This study demonstrated that patellar morphology differs significantly between domestic cats and dogs, both in terms of shape and size. Dogs exhibited a higher degree of morphological variation, which may be linked to breed diversity and functional adaptation. The primary axis of shape variation (PC1) effectively distinguished the two species, with shape trends supported by strong statistical results. A significant allometric effect was also identified, indicating that size plays a major role in shaping patellar morphology. These findings suggest that using a single standardized surgical approach or implant design may not be optimal for all individuals, particularly in dogs. Three-dimensional geometric morphometrics provides a valuable framework for quantifying anatomical variation, and future studies incorporating breed-specific analysis may further support the development of more personalized, anatomically adapted solutions in veterinary orthopedic surgery.

## Data Availability

The data presented in this study are available upon request from the corresponding author (O.G.).
